# Draft Genome Sequence of *Pseudomonas nitroreducens* Strain TX1, Which Degrades Nonionic Surfactants and Estrogen-Like Alkylphenols

**DOI:** 10.1128/genomeA.01262-13

**Published:** 2014-01-30

**Authors:** Shir-Ly Huang, Hsin Chen, Anyi Hu, Nguyen Ngoc Tuan, Chang-Ping Yu

**Affiliations:** a Department of Life Sciences, National Central University, Jhongli, Taiwan; b Key Laboratory of Urban Environment and Health, Institute of Urban Environment, Chinese Academy of Sciences, Xiamen, China

## Abstract

*Pseudomonas nitroreducens* TX1 ATCC PTA-6168 was isolated from rice field drainage in Taiwan. The bacterium is of special interest because of its capability to use nonionic surfactants (alkylphenol polyethoxylates) and estrogen-like compounds (4-*t*-octylphenol and 4-nonylphenol) as a sole carbon source. This is the first report on the genome sequence of *P. nitroreducens*.

## GENOME ANNOUNCEMENT

Octylphenol polyethoxylate (OPEO_n_) and nonylphenol polyethoxylate (NPEO_n_) are nonionic surfactants extensively used as detergents, emulsifiers, and dispersants (1, 2). Nonylphenol, 4-*t*-octylphenol, and carboxylated intermediates are known metabolites from these surfactants (3, 4). Two enzymes, OPEO_n_ alcohol dehydrogenase (*Pseudomonas putida* S-5) and NPEO_n_ alcohol dehydrogenase (*Ensifer* sp. strain AS08), are reported to be able to shorten the ethoxylate chains (5, 6). The other growth carbon sources, 4-*t*-octylphenol and 4-nonylphenol, are commercial products and endocrine disruptors (7–9). The ubiquity of alkylphenols in environments has been investigated (3, 9–11). One of the proposed mechanisms for bacterial disruption of estrogen activity is involved in *ipso* substitution catalyzed by single-component monooxygenase in strains of *Sphingomonas* spp. (12–14). The other mechanism is related to the mono-oxygenation of the phenol ring by multicomponent phenol hydroxylase (15–19) or by cytochrome P450 monooxygenase (20), followed by aromatic ring-cleavage (19). The transporters for these carbon sources so far are unknown.

*P. nitroreducens* TX1 was isolated from the sediment in rice field drainage (4, 17, 19, 21–23). The bacterium was demonstrated to be able to use OPEO_n_ (Triton X-100; average n, 9.5), and NPEO_n_ (Triton N-101; average n, 9.5). It was demonstrated that it grows on minimal basal salts medium containing 0.05% to 20% OPEO_n_ and shortens the ethoxylate chain, and then it produces octylphenol (4). In addition, the strain also grows on 4-*t*-octylphenol and 4-nonylphenol as a sole carbon source (17). Strain TX1 is the first bacterium that is able to degrade both OPEO_n_/NPEO_n_ and 4-*t*-octylphenol/nonylphenol.

The genome of *P. nitroreducens* TX1 was sequenced by a whole-genome shotgun strategy using Solexa HiSeq 2000 paired-end sequencing and assembled *in silico* using SOAP*denovo* (version 1.05); this resulted in 138 contigs (>200 bp in size) with an N_50_ length of 111,179 bp. The protein-encoding genes were predicted using Glimmer 3.02 (24), tRNAscan-SE (25), and RNAmmer (26). The genome sequences were also annotated by Rapid Annotations using Subsystems Technology (RAST) (27). The functions of the predicted coding sequences (CDSs) were then annotated in NCBI-NR (28), COG (29), and KEGG (30). The draft genome sequence of strain TX1 has a total of 6,700,249 bp, with a G+C content of 64.5%. It contains 6,341 CDSs, one 16S-23S-5S operon, and 50 tRNAs. Of the predicted proteins, 87.3% were classified into 23 COG categories.

For the genes that may be involved in ethoxylate chain degradation, two quinoprotein alcohol dehydrogenase genes, two aldehyde dehydrogenase genes, and a pyrroloquinolone quinine biosynthesis cluster were shown to be clustered and to be upregulated when the cells were grown on OPEO_n_. The genes for the oxygenase component of cytochrome P450 monooxygenase and catechol 1,2-dioxygenase were identified. Genes encoding a multicomponent phenol hydroxylase and catechol 2,3-dioxygenase were shown to be clustered. They might be involved in the alkylphenol degradation. Three porins and four ABC-type transporters are upregulated and may be related to the transportation of nonionic surfactants.

### Nucleotide sequence accession numbers.

The draft genome sequence of *P. nitroreducens* strain TX1 has been deposited at GenBank under the accession no. AMZB00000000. The version described in this paper is the ﬁrst version, AMZB01000000.
